# Household

**Published:** 1887-01

**Authors:** 


### HOUSEHOLD.

FRESH MEAT.—Never allow fresh meat to remain in paper; it absorbs the juices.

LEMON PUDDING.—Three eggs, one scant cup of sugar, two liberal tablespoonfuls of corn-starch, one lemon, juice and rind, two cups of milk, one heaping teaspoonful of butter. Scald the milk, and stir in the corn-starch wet up in four teaspoonfuls of cold water. Cook—stirring all the time—until it thickens well, add the butter, and set aside until perfectly cold. Then beat the eggs light, add the sugar, the lemon juice and grated peel, and whip in, a great spoonful at a time, the stiffened corn-starch mild. Bake in a buttered dish, and eat cold.

DELICIOUS PUDDING.—A delicious pudding is made by stirring one pint of thick, sweet cream with three ounces of sugar and the yolk of three eggs. Sprinkle fine bread crumbs over the bottom of a pudding dish, then put the cream over it, and a thin layer of bread crumbs on the top ; bake this for half an hour in a hot oven. Make a frosting of the whites of the eggs with sugar enough to make it stiff ; before spreading this on the top of the pudding, lay thin slices of orange all over the top. Cover this with the frosting.

TO MAKE CHOCOLATE.—A dainty way to make chocolate is to set an earthen pot into a kettle of boiling water ; in this place a quart of milk and cream mixed. Stir into this when it is hot a paste made by mixing three heaping tablespoonfuls of grated chocolate with a little cold milk. Let this boil for two or three minutes, and serve very hot. If the chocolate is sweetened, it is better not to add sugar, but to let each one add it at the table if it is not sweet enough, but if the unsweetened chocolate is used, two dessertspoonfuls may be put in while it is cooking. Cream is the greatest possible addition.

EGGS ON TOAST.—Six eggs, one cupful drawn butter—drawn in milk ; slices of stale bread, toasted and buttered ; chopped parsley ; pepper and salt. Heat a cupful of milk to scalding ; mix in a large teaspoonful of butter, a tablespoonful of flour wet with cold water and rubbed smooth, and stir until it is as thick as custard. Add chopped parsley, pepper and salt to taste. All this should be done in a tin vessel set in boiling water, and over the fire. Have ready the toast, not forgetting to pare the crust from each slice before it is toasted, buttered, and laid in close rows upon a hot dish. Pour a tablespoonful of hot water on each piece. Beat the eggs very light, and stir first into drawn butter until they are a rich, yellow sauce, almost stiff enough to stand alone. Heap upon the toast, and send hot to table.

TO REMOVE INK STAINS.—A solution of oxalic acid has been used for removing ink stains from cotton, linen or the fingers, but it is attended with the danger of injuring textiles and the skin. A much safer and better treatment of ink or rust stains consists of the application of two parts of powdered cream of tartar and one part of finely powdered oxalic acid. Shake up the ingredients well together and apply the powder with a dry rag to the dampened stain. When the spot has disappeared the part should be well washed. T remove ink stains from paper make a solution of fresh muriate of tin, two drams; water, four drams; and apply with a camel's hair brush.

Fresh Eggs.—Never place fresh eggs near lard, fruit, cheese, fish or other article from which any odor arises. The eggs are extremely active in absorbing power, and in a very short time they are contaminated by the particles of objects in their neighborhood, by which the peculiar and exquisite taste of a new-laid egg is destroyed.

Cure For Sprain.—Take one tablespoonful of honey, the same of salt, and the white of one egg; beat all well together for at least one hour, or two would be better. Let it stand an hour. Then anoint the sprained part freely; keep well rolled up with a good bandage.

Tea.—Never make tea in a tin pot. The tannin, which is acid, attacks the tin and produces a poison.

Hotch-potch is an old-fashioned Scotch dish, made in the spring, when there are plenty of fresh vegetables. It is a thick puree-like soup. It may be made either from fresh or cooked meat. This is one way of making it: three or four pounds of loin chops are put into a saucepan with about three quarts of boiling water. Peas, haricot beans, carrots, half a turnip, parsley, a little bit of cabbage. and some green onions are added. Boil this very slowly for an hour and a quarter, season with pepper and salt. It should be a thick broth when done.—*Correspondent London Queen.*

White Lemon Cream.—Boil the thin peel of two lemons in one pint of cream, strain, and thicken with the well-beaten yolks of three and the whites of four eggs, into which half a tea-cupful of white sugar has been beaten. Add half a saltspoonful of salt, stir rapidly with the egg-beater until nearly cold, and pour it into glasses or cups. This quantity will fill six custard cups.

Milk Toast.—Wet the pan to be used with cold water, which prevents burning. Melt an ounce of floured butter; whisk into it a pint of hot milk; add a little salt; simmer. Prepare four slices of toast, put them in a deep dish one at a time, pour a little of the milk over each, and over the last one pour the remainder of the milk.

Boiled Indian Pudding.—One cup sweet milk, part cream, sour milk or buttermilk; three tablespoonfuls of molasses, one teaspoonful of salt, one teaspoonful of saleratus, one cup of meal, one cup of flour. Dried fruit if you like. Steam 1½ hours.

Jumbles.—One cup of butter, two cups of sugar, three eggs, one teaspoonful of soda, two of cream tartar, two tablespoonfuls of warm water to dissolve the soda. No other wetting is used, but the dough is made very stiff and rolled out thin.

Cheap Tea Cake.—One cup of sugar, one cup of milk, three cups of flour and one-half cup of butter, two teaspoonfuls of baking powder, one teaspoonful of caraway seeds and two tablespoonfuls of currants.

Boiled Tongue.—Soak it all night before using it, and be careful to wash out all the salt which is put into various crevices to preserve it. Boil in plenty of water till tender. Remove the skin before sending to the table and garnish with parsley.

Fried Potatoes.—Pare some potatoes to the shape of a ball, cut each ball in six pieces to resemble the quarters of an orange, chamfer the edges slightly. Dry them effectually in a napkin, put them in a frying basket and plunge it in boiling fat; keep shaking the basket until the potatoes assume a golden color. Turn them out on a cloth in front of the fire to drain and sprinkle them freely with fine salt.

